# Sorption Kinetics on Open Carbon Nanohorn Aggregates: The Effect of Molecular Diameter

**DOI:** 10.3390/molecules21040521

**Published:** 2016-04-21

**Authors:** Brice A. Russell, Pravin Khanal, Maria M. Calbi, Masako Yudasaka, Sumio Iijima, Aldo D. Migone

**Affiliations:** 1 Department of Physics, Southern Illinois University, Carbondale, IL 62901, USA; bricer@siu.edu (B.A.R.); khanal@siu.edu (P.K.); 2 Department of Physics & Astronomy, University of Denver, Denver, CO 80208-6900, USA; Maria.Calbi@du.edu; 3 National Institute of Advanced Industrial Science and Technology, Tsukuba 305-8565, Japan; m-yudasaka@aist.go.jp; 4 Graduate School of Science and Technology, Meijo University, Shiogamaguchi, Tempaku, Nagoya 468-8502, Japan; chie@ccmails.meijo-u.ac.jp

**Keywords:** adsorption, sorption, kinetics of adsorption, Carbon Nanohorns, adsorption isotherm, linear driving force model

## Abstract

We present the results of a study of the kinetics of adsorption on aggregates of open carbon nanohorns using argon and CF_4_ sorbates. We measured the equilibration times for each value of the sorbent loading along eight adsorption isotherms (four isotherms for each sorbate species). We found that: the equilibration times decrease as the sorbent loading (and the equilibrium pressure of the coexisting gas) increases, for a given temperature; and, that, for a given value of the sorbent loading, the equilibration times decrease with increasing temperature. When considering the effect of scaling of the temperatures by the respective critical temperatures we found that, at the same scaled temperature and at comparable loadings, the equilibration times for CF_4_ were longer than those for argon. We discuss a possible explanation for this result.

## 1. Introduction

Carbon-based sorbents are of great importance, both from a fundamental, as well as from a practical perspective. Studies of adsorption on exfoliated graphite [1], and, on both single-walled carbon nanotubes [2,3] and nanotube bundles [4,5,6], have provided excellent experimental realizations of matter that are effectively two- and one-dimensional (respectively). On the other hand, adsorption on activated carbons [7] has numerous industrial applications in purification and storage.

Studies of adsorption on various forms of single-wall carbon have been consistently garnering the attention of researchers for a number of years [2,3,4,5,6,8]. Carbon nanohorns are a form of single-walled carbon that was discovered in 1999 by S. Iijima [9]. Depending on the conditions under which the carbon nanohorn aggregates are synthesized, they can be of one of four different types: dahlia-like [9,10,11], bud-like [10], petal-like [11], or seed-like [12]. Unlike single-walled carbon nanotubes (which aggregate forming cylindrical bundles, along the long axes of the nanotubes), carbon nanohorns aggregate forming sphere-like arrangements [9,10,11,12].

**Figure 1:**
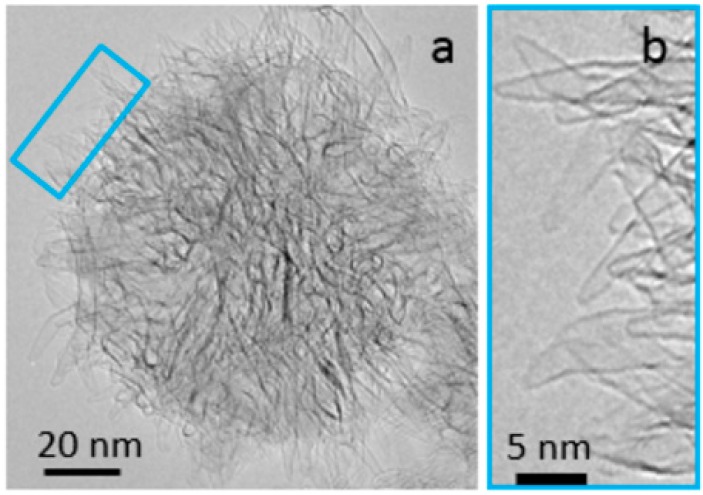
TEM images of carbon nanohorns. (**a**) a dahlia-like aggregate; and (**b**) the magnified image of the individual nanohorn tips. The figure is reproduced from reference [13].

In the dahlia-like aggregates [9,10,11], the type of nanohorns used in this study, the tips of the individual nanohorns (the “horns”) protrude from the surface of the aggregate; this is not the case for the other types of nanohorn aggregates. Figure 1 displays TEM images of a dahlia-like carbon nanohorn aggregate, from reference [13].

Individual nanohorns were isolated from dahlia-like aggregates only about a decade after the discovery of this form of carbon [14]. It was found that the vast majority of the individual nanohorns were either two-lobed (*i.e.*, nanotube-like, with two horn ends) or three-lobed (like a three-point ninja star, with three ends). Individual carbon nanohorns are closed, single-carbon, structures that end in roughly conical tips (the “horns”). The diameters of the lobes range between 2 and 10 nm [14]. The lobes of the individual nanohorns are not uniform in diameter along their lengths; they are irregular. The lengths of the lobes range between 10 and 70 nm [14]. The diameters of the sphere-like nanohorn aggregates, typically, range between 80 and 120 nm [9,14]. Roughly, we can describe individual carbon nanohorns as resembling a much shorter and wider, more defected, and much less uniform, version of single-walled nanotubes or of branched nanotubes.

Nanohorns (like individual single-wall nanotubes) are closed. The space at the interior of the nanohorn is not accessible for sorption, unless the single-carbon wall that forms it has been chemically opened [9,15]. One practical advantage of the lower degree of uniformity exhibited by carbon nanohorns (when compared to single walled nanotubes) is that they are relatively easier to open: milder chemical treatments are effective in opening carbon nanohorn aggregates [15].

Due to the more or less radial arrangement of the nanohorns in the aggregates, two groups of adsorption sites can be identified in aggregates of closed carbon nanohorns [16,17,18]. One group are the high energy binding sites, in which the sorbate species is attracted by C atoms from more than one nanohorn. These sites are in the deeper portions of the more or less conical interstitial spaces that exist between the lobes of three or more different nanohorns, each of which points more or less radially out from the center of the aggregate [18]. The other group are the low energy binding sites where, effectively, the sorbate is attracted only by carbon atoms from one nanohorn. These sites are closer to the outer edges of the spherules, where the carbon nanohorns are sufficiently far apart that a sorbate molecule is only attracted by C atoms from one nanohorn, because the other nanohorns are too far away to have much of an effect [18]. It is important to note that a significant difference exists in adsorption in the interstitials between aggregates of nanohorns and bundles of closed nanotubes: while the interstitial spaces in the nanohorn aggregates are accessible to many sorbates [18] (owing to the generally open, more or less conical, nature of these spaces), it has been shown that the interstitial spaces in the carbon nanotube bundles are such that they cannot be occupied by any species [19,20].

The same two groups of adsorption sites are available for chemically-opened nanohorn aggregates. In addition, on open nanohorns the space at the interior of the individual nanohorns is also available. The interior space also has high energy binding sites: the spaces close to the interior of the tips or horns of the lobes of the nanohorns; and low energy binding sites: the regions away from the tips [16,17,21]. Experimentally, it has been found that the external and interior high energy binding sites have comparable energies, as do the external and interior low energy binding sites since only two groups of adsorption energies can be resolved in the isotherms (we note, however, that nanohorn aggregates are rather energetically inhomogeneous systems) [21].

In adsorption, it is almost always the case that there are more studies of the equilibrium thermodynamic properties of systems than there are of those involving the kinetics of adsorption (*i.e.*, how equilibrium is reached) [22,23]. This, in spite of the fact that practical applications depend both on the equilibrium properties and on the kinetics of adsorption. This is also the case for adsorption studies on carbon nanohorns. We are aware of only one other study of the kinetics of adsorption on carbon nanohorn sorbents [21], and of none that have explored the dependence of the sorption kinetics on temperature or sorbate size. Most adsorption studies on carbon nanohorns have focused on the equilibrium properties of the sorbed system (see, for example, [24,25,26,27,28]).

Here we address the question of the kinetics of adsorption on aggregates of chemically-opened carbon nanohorns. We report on the results of a study of the kinetics of adsorption of two sorbates: argon and carbon tetrafluoride. The results obtained allow the determination of the effect of mass loading, temperature, and, sorbate molecular size on the kinetics of adsorption on opened nanohorns.

## 2. Results

The kinetic measurements were taken along adsorption isotherms conducted at eight different temperatures (four temperatures for each of the sorbate species). The sorbent was not evacuated after each dose of gas reached equilibrium along an isotherm; rather, more gas was added to the cell in order to increase the loading of the sorbent at equilibrium (for the next point along the isotherm). The pressure of gas inside the cell was measured and recorded at specified intervals (typically, every 10 s). The loading of the carbon nanohorn sorbent was obtained from it.

### 2.1. Argon

**Figure 2:**
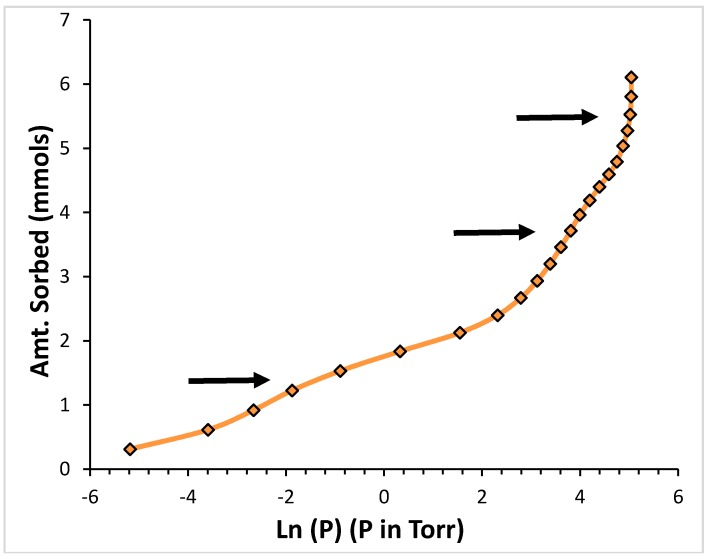
Adsorption isotherm for Ar on the chemically-opened nanohorns at 76.32 K. The arrows indicate (from the lowest to the highest) adsorption on the high energy binding sites, the low energy binding sites, and the saturated vapor pressure. The amount sorbed is in millimoles.

Figure 2 shows the adsorption isotherm for Ar at 76.32 K. The data are presented in a logarithmic plot.

The logarithm of the pressure is directly proportional to the chemical potential of the sorbent in the gas phase (assumed to behave as an ideal gas) [29]. Owing to the equilibrium condition between film and gas, the chemical potential of the gas and that of the sorbed film are equal [30]. Thus, regions of steep increase in loading with little change in the logarithm of the pressure in the isotherm correspond to regions of little change in the energy of the sorbed species [30]. These sections in the isotherm allow us to identify adsorption occurring on regions of similar binding energy. Sorption on the high binding energy sites are indicated by the arrow at the lowest pressures and loadings; sorption on the low binding energy sites, by the arrow at the intermediate pressures and loadings; and the system reaching the saturated vapor pressure (*i.e.*, the formation of the condensed bulk phase) is indicated by the arrow at the highest pressures and loadings [21]. Our kinetic measurements were conducted for each point along the isotherm, sequentially, from the lowest to the highest loadings.

**Figure 3:**
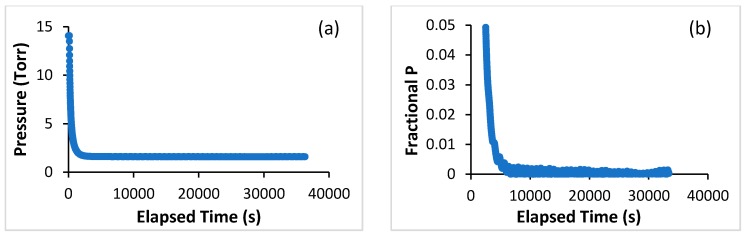
Approach to equilibrium for point 6 along the 82.07 K isotherm for Ar on the open nanohorns (the point numbers start at 1 with the point at the lowest loading, 2 for the next one, *etc.*). (**a**) Absolute pressure (in Torr, Y axis) as a function of the time elapsed (in seconds) since a dose of gas was added; and (**b**) fractional pressure distance to equilibrium [(P(t) − P_eq_)/P_eq_] as a function of time elapsed since dosing (data shown for (**b**) start ~40 min after the dose was added). From (**b**) it is clear that the noise level in the pressure is reached only after ~2 h have elapsed.

After a dose of sorbate is introduced into the experimental cell, there is an initial period of rapid decrease in the pressure inside the cell. This is followed by a much slower pressure decrease until equilibrium is reached. This is shown in Figure 3, which displays the decrease in the pressure in the sample cell as a function of time for one point (point 6) along the 82.07 K isotherm for Ar, both in terms of the absolute pressure (Figure 3a), as well as in terms of the fractional pressure distance to equilibrium(Figure 3b). This latter quantity is defined as: ΔP/P_eq_ where ΔP = P(t) − P_eq_ with P_eq_ being the equilibrium value of the pressure, and P(t) the value of the pressure at time t after the gas dosed into the cell. Equilibrium, for either Figure 3a,b, corresponds to the pressure or fractional pressure becoming a horizontal line as a function of time.

For the data displayed in Figure 3, the fractional approach to equilibrium in plot (b) shows that 99% of the fractional pressure change occurs within the first hour, or less, after the gas is added to the sample cell. The plot, in (b), also shows that two hours or more need to elapse in order for equilibrium to be reached.

We computed the mass sorbed in the open carbon nanohorn aggregates as a function of time elapsed after the gas dose was added, for each point along the isotherm with the same expression that we use to compute the mass loading at equilibrium.

We have compared our mass loading data to the linear driving force model [22,23]. The expression for the mass loading in the linear driving force model is:

(M(t)/M_eq_) = [[1 − exp(−kt)] (1)

**Figure 4:**
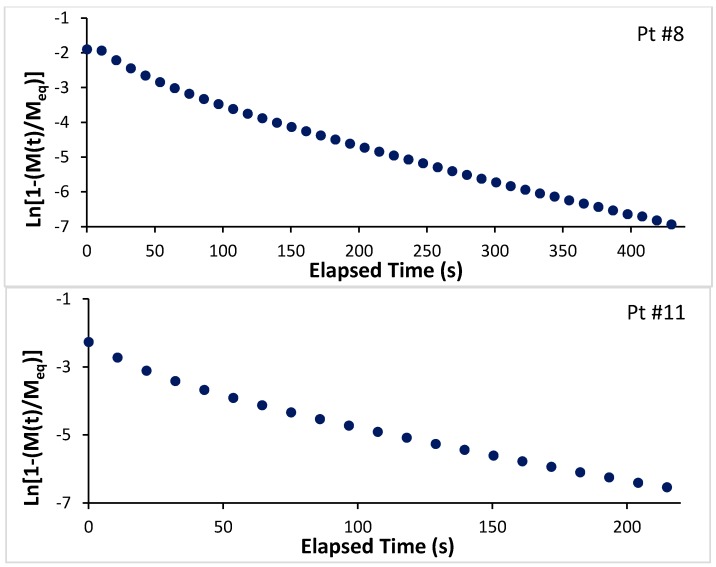
Mass loading as a function of time elapsed using an expression derived from Equation (1) for two points along the 82.07 K isotherm (top panel for point 8 along the isotherm; bottom panel for point 11 along the isotherm). If the linear driving force model were to describe the data accurately, the plots should be straight lines. With the exception of the initial times, the linear driving force model does an adequate job of describing the data.

We note that the data in Figure 4 is reasonably well-described by the above expression, and that the deviation is more noticeable at the initial times.

It is clear from Figure 4 that the kinetics of adsorption become faster with increasing sorbent loading: point 11 (measured later in the isotherm) corresponds to a higher equilibrium loading on the sorbent than point 8, and it takes approximately half the time for point 11 to reach the same fractional change in loading than it does for point 8 (the time scale for the bottom plot is only half that of the top plot). This can also be re-stated by saying that the kinetics of adsorption is faster when the equilibrium pressure in the gas phase increases.

**Figure 5:**
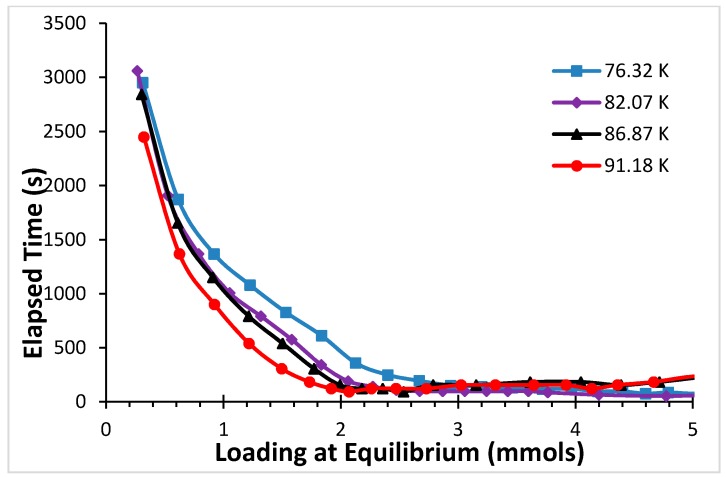
Time required to reach 99% of the equilibrium mass loading (in seconds) as a function of the mass loading at equilibrium for the four temperatures studied 76.32 (**blue**), 82.07 (**purple**), 86.87 (**black**), and 91.18 K (red) for argon on aggregates of open nanohorns.

While it is clear that in every case we have waited sufficiently to reach equilibrium (see Figure 3), there is considerably more noise in the determination on the first time that equilibrium is reached in the mass loading than there is in determining the first time that 99% of the equilibrium mass loading value is reached. This is why we have chosen the latter to determine the trends in the behavior of the kinetics of adsorption of the system. We have determined the dependence of the equilibration time on equilibrium loading by measuring the time required for reaching 99% of the equilibrium mass loading after a dose of gas is added to the cell as a function of the total equilibrium loading. These results are displayed in Figure 5.

### 2.2. CF_4_

We have performed measurements of the adsorption kinetics for CF_4_ at four temperatures on the same sample of aggregates of open carbon nanohorns on which the argon measurements were conducted. The temperatures studied were 108. 07, 118.95, 130.87, and 141.85 K.

**Figure 6:**
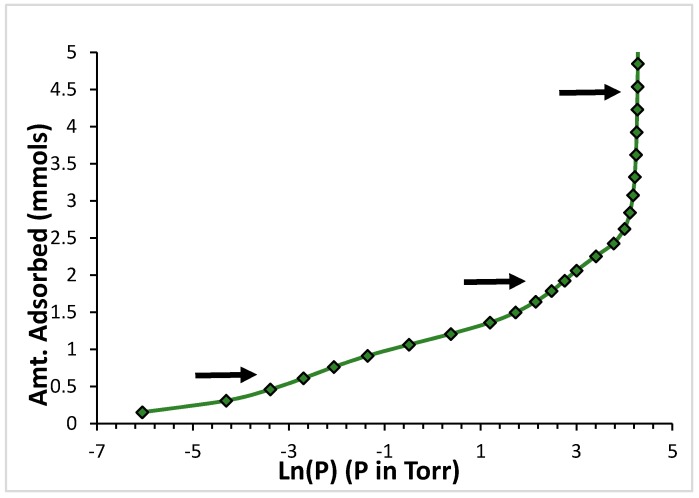
Adsorption isotherm for CF_4_ on aggregates of open carbon nanohorns at T = 118.95 K. The arrows, from top to bottom, indicate saturation, sorption on weak binding energy sites, and sorption on strong binding energy sites present on the sorbent.

The isotherm features for the CF_4_ data are very similar to those shown in Figure 2 for Ar. Figure 6 presents a logarithmic plot the adsorption isotherm data for CF_4_ at 118.95 K. The same three regions of steep loading increasing present in Figure 2 for Ar are present also for CF_4_, and they have the same origin: the lower-loading, lower-pressure sub-step corresponds to sorption occurring in the higher binding energy sites present in the aggregates; the sub-step at intermediate pressures and loadings corresponds to sorption occurring in the lower energy binding sites; and, the near vertical step at the highest pressures corresponds to the system reaching the saturated vapor pressure.

We conducted the same analysis of the CF_4_ kinetic data that we did for argon, so we will not repeat it here. Since we have described it already in some detail, rather than repeating it, we proceed to present a graph that summarizes our results for CF_4_.

**Figure 7:**
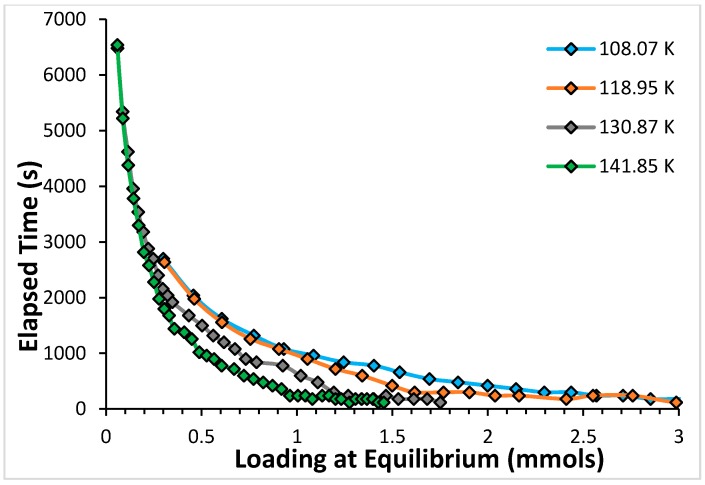
Equilibration times, in seconds, are measured at the point when the sorbent loading reaches 99% of the sorbent loading at equilibrium for the four temperatures studied, as a function of the loading at equilibrium for CF_4_.

Figure 7 displays the time required to reach equilibration, evaluated at the time when mass loading has reached 99% of the equilibrium mass loading value for the point, as a function of the equilibrium mass loading, for the four temperatures studied for CF_4_.

## 3. Materials and Methods

We used a specialty-built adsorption setup to conduct the kinetic of adsorption measurements [31]. Low temperatures were achieved through the use of a helium closed-cycle refrigerator and a two-stage temperature control setup that allows regulation of the temperature to within ±20 mK, as determined from measurements at the saturated vapor pressure. The pressures were measured using capacitance manometers with maximum pressure ranges of 1, 10, and 1000 Torr. The pressures were recorded using an in-house written LabView program, which was also used for dosing the gas into the experimental sample cell [31].

In the adsorption experiments, a known amount of gas was admitted into the cell containing the carbon nanohorn sample. The drop in pressure that follows the dosing of the gas was monitored as a function of time as the gas adsorbed onto the sample until the pressure reached a constant value, within noise levels. We stayed at this constant value of the pressure for an amount of time on the order of several hours. This final pressure was recorded as the equilibrium vapor pressure and the amount adsorbed at that point was calculated from it. This process of adding a known amount of gas and waiting for the next equilibrium pressure and loading to be reached was repeated until the saturated vapor pressure of the gas/adsorbate was reached. The final equilibrium pressure and the amount adsorbed at each of these points are then plotted as an adsorption isotherm.

The carbon nanohorn sample was both produced and treated at the Japan Science and Technology Agency. The mass of the carbon nanohorn sample used in the experiments was 0.1692 g. The nanohorns were subjected to an oxidation treatment in air for 9 h in which the temperature increased at a rate of 1 °C/min from room temperature to 550 °C followed by natural cooling back to room temperature in order to open them. This slow-heating treatment has been reported to result in the formation of a small number of carboxyl groups on the nanohorns [32], which do not significantly affect the adsorption in the interior spaces of the nanohorns (an image of an individual as-produced and one of a chemically-opened nanohorn can be found in Figure 1 of reference [33]).

The gases used were research purity gases from Matheson Tri-Gas (Waverly, TN, USA).

## 4. Discussion

It is important to discuss whether the approach used here, that of determining the length of time needed to reach 99% of full loading, is an appropriate way to determine the dependence of equilibration times on loading. This approach is based on the supposition that the behavior of the equilibration time of a system as a function of equilibrium loading is the same at 99% of full loading than it is at full equilibrium. For CF_4_ and Ar it turns out that this is indeed the case. We know this to be so because we have data extending up to equilibrium loading for all points. However, this is not the case for all sorbates. For other systems, such as ethane [34], there is a reversal in the dependence of the time needed to reach equilibrium on loading determined using data closer to equilibrium than 99% as opposed to data at 99% of equilibrium loading. The only sure way to establish how equilibration times depend on loading is by conducting measurements up to the point when the system has reached equilibrium.

Figure 5 presents a summary of our results for argon. The data displayed in this figure allows us to determine the effect of both mass loading (or, alternatively, of the equilibrium pressure value of the coexisting gas phase), as well as that of temperature in the equilibration times. As the data in Figure 4 clearly shows, in the region where the high binding energy sites (interstitials and close to the inside of the horns) are being occupied (at low loadings and low equilibrium gas pressures), the equilibration times are much longer than on the low energy binding sites, which are occupied at higher equilibrium gas pressures and higher loadings. The data also show that, for a given sorbent loading, equilibrium is reached faster at higher temperatures.

The data for CF_4_ displayed in Figure 7 show that, just as the case for argon, for a given temperature, equilibration times decrease with increasing loading (or increasing equilibrium gas pressure in the cell); and, for a given sorbent loading, they decrease with increasing temperature also for this sorbate.

We have data at four different temperatures for argon: 76.32, 82.07, 86.87, and 91.18 K. For reference, argon has a critical point of 150.687 K. In order to compare our results for argon to those for CF_4_, we scaled the measurement temperatures by the critical temperature, T/T_c_. The argon experiments, hence, were performed at the following scaled temperatures: T/T_c_ = 0.506, 0.544, 0.576, and 0.605. The critical temperature for CF_4_ is T_c_ = 227.7 K. So, scaling the experimental temperatures (*i.e.*, 108.07, 118.95, 130.87, and 141.85 K) by the critical temperature (T/T_c_) results in the following set of scaled temperatures for CF_4_: 0.475, 0.522, 0.575, 0.623. The set of scaled temperatures for CF_4_ encompasses that which was covered in the argon measurements.

The data presented for argon in Figure 2 corresponds to T/T_c_ = 0.506, very close to the scaled temperature value of T/T_c_ = 0.522 for the isotherm displayed for CF_4_ in Figure 6. It is interesting to consider the effect of scaling of the temperatures by the respective critical temperatures. We compare the features displayed in these two isotherms. They occur at, roughly, the same values of the pressure (the lower pressure sub-step for both sorbates occurs near −2.5; the intermediate pressure sub-step for both sorbates occurs near +3). This suggests that the ratios of the sorbate-sorbent interaction energy to the respective absolute isotherm temperatures must be roughly comparable.

Figure 5 for argon and Figure 7 for CF_4_, both of which represent a summary of the kinetics results for the respective systems, have qualitatively the same features.

We note that the sorbent loading data is presented in absolute units in both Figure 2 and Figure 6. The molecular size of argon differs from that of CF_4_. Rather than comparing the equilibration times at the same absolute loading for these two different sorbates, it is more appropriate to compare equilibration times at corresponding points along the isotherm (these points correspond to the same degree of occupation of the sorbent, which corresponds to different absolute number of sorbate molecules for each species).

Comparing Figure 2 and Figure 6 we note that the top of the high energy binding sites corresponds to ~0.76 mmols for CF_4_ and to ~1.5 mmols for argon. Similarly, the top of the low energy binding sites for CF_4_ in Figure 6 is at ~2.2 mmols, while in the case of argon is at ~4.2 mmols. There is, roughly, a factor of two in the number of mmols required to fill the sorption sites on the open carbon nanohorns with argon and with CF_4_. Taking this factor into account when we compare the results from Figure 5 and Figure 7, it is clear that the equilibration times are longer for CF_4_ than they are for argon at loadings corresponding to the same degree of occupation of the sorbent.

As was discussed before, the temperatures have been selected so that the ratio of the binding energies to the measurement temperatures should be comparable. Thus, the difference in binding energies between argon and CF_4_ in aggregates of carbon nanohorns should not be the main factor explaining this observed difference in equilibration times.

In the case of spherical and effectively spherical sorbates on a planar sorbent, the times to equilibration decrease when the gas pressure increases [35]. Since the pressures at which all the isotherm features occur are roughly the same, and since the temperatures are scaled temperatures, we could have, naively, expected similar equilibration times in this case as well. This naïve expectation is incorrect, however, because the open carbon nanohorn aggregates are far from a planar sorbent: aggregates of open nanohorns have a significant portion of porous sorption sites in them.

The slowing down that we are measuring in the sorption kinetics between these two sorbates under these scaled conditions is most likely the result of the difference in their molecular sizes. CF_4_ (which at the temperatures in question will be effectively a sphere) has a considerably larger diameter than argon. Hence, CF_4_ also will also have greater difficulty than the smaller argon in accessing all the available sorption sites in the interstitials, and in penetrating into the interior of individual nanohorns through openings in the chemically-treated material. Our scaled data allows us to separate the effect of molecular diameter in the sorption kinetics for this sorbate.

## 5. Conclusions

The kinetics of adsorption were explored for Ar and CF_4_ adsorption on oxidized carbon nanohorns.

The kinetics of adsorption for argon obey a linear-driving-force model for sorbent loading at all but the smallest values of time. The evolution of the pressure and the mass loading data for CF_4_ is qualitatively similar to that of Ar.

In order to obtain general trends in the adsorption kinetics behavior as a function of loading, sorbate molecular size, and, temperature, we measured the time it took to reach 99% of the equilibrium coverage for each point along the four isotherms for both Ar and CF_4_. Values at 99% of equilibrium coverage were chosen in order to extract trends in the data because for values closer to 100% of the equilibrium coverage, the pressure data shows considerable noise. We note that, although it is not always the case for all sorbate-sorbent combinations, for Ar and CF_4_ adsorption on oxidized carbon nanohorns the time to reach 99% equilibrium coverage follows the same overall trend observed at 100% equilibrium coverage.

Both for argon and for CF_4_, the time to reach equilibrium decreases as both isotherm temperature and coverage increase. The difference between the kinetics of adsorption for Ar and CF_4_ are in the time that each species takes to reach equilibrium at comparable fractional coverages and reduced temperatures. CF_4_ takes on the order of twice the amount of time to come to equilibrium at the same value of the comparable loading and scaled temperature. We attribute this difference in behavior to the larger size of the roughly spherical CF_4_ molecule. Larger molecular sizes make it is more difficult for this sorbate to reach equilibrium in the porous regions of the oxidized carbon nanohorns relative to argon.

Methods such as those described in this paper can be used to experimentally investigate the mechanisms governing the speed with which gases are taken up on, and in, porous materials. Studies are ongoing to elucidate the effects of size and shape on the kinetics of adsorption on this sorbent.
